# Is the Mitochondrial Function of Keloid Fibroblasts Affected by Cytoglobin?

**DOI:** 10.21315/mjms2021.28.2.4

**Published:** 2021-04-21

**Authors:** Sri Widia A Jusman, Isma Nur Azzizah, Mohamad Sadikin, Novi Silvia Hardiany

**Affiliations:** 1 Department of Biochemistry & Molecular Biology, Faculty of Medicine Universitas Indonesia, Jakarta, Indonesia; 2 Master’s Program in Biomedical Sciences, Faculty of Medicine Universitas Indonesia, Indonesia; 3 Center of Hypoxia & Oxidative Stress Studies, Faculty of Medicine Universitas Indonesia, Jakarta, Indonesia

**Keywords:** CYGB siRNA, PGC-1α, SDH enzyme activity, keloid fibroblasts

## Abstract

**Background:**

A keloid is a benign skin tumour characterised by excessive proliferation of fibroblasts, a process that requires a sufficient amount of energy. The energy needs are associated with adequate oxygen (O_2_) flow and well-functioning mitochondria. It is known that cytoglobin (CYGB) has a function in O_2_ distribution. The aim of the present study was to explore whether the inhibition of CYGB expression caused impaired mitochondrial function of keloid fibroblasts.

**Methods:**

An in vitro study was conducted on a keloid fibroblast derived from our previous study. The study was carried out in the laboratory of the Biochemistry & Molecular Biology Department, Faculty of Medicine, Universitas Indonesia (FMUI), from July to December 2018. CYGB expression was inhibited by small interfering ribonucleic acid (siRNA) and CYGB. Analysis of mitochondrial function was observed through peroxisome proliferator-activated receptor gamma coactivator-1α (PGC-1α), a mitochondrial biogenesis marker and the activity of the succinate dehydrogenase (SDH) enzyme in mitochondria.

**Results:**

The CYGB gene and protein were downregulated after treatment with CYGB siRNA. Inhibition of CYGB expression with siRNA also tended to decrease the levels of PGC-1α messenger ribonucleic acid (mRNA) and protein, as well as SDH enzyme activity.

**Conclusion:**

Inhibition of CYGB expression with siRNA tended to decrease mitochondrial biogenesis and function. This may be useful for understanding the excessive proliferation of fibroblasts in keloids and for development of treatment for keloids.

## Introduction

Fibroblasts are the main cellular component of connective tissue, which plays a role in the synthesis of extracellular matrix proteins. Activated fibroblasts are mostly found in keloids (1). Keloids are benign tumours of the skin that arise owing to excessive fibrogenesis in wound healing. Collagen synthesis by keloid fibroblasts increases 12–20 times compared to normal tissue. Once fibroblasts are activated, there is an increase in proliferation, resulting in hypoxia (2), a condition in which the oxygen concentration in the tissue is below the normal range. Cells in hypoxic conditions will try to increase adenosine 5′-triphosphate (ATP) production through mitochondrial biogenesis. Mitochondrial biogenesis can be assessed through the expression of peroxisome proliferator-activated receptor gamma coactivator-1α (PGC-1α), which is a regulator of mitochondrial biogenesis (3). PGC-1α protein acts as a coactivator of several transcription factors involved in metabolic processes such as gluconeogenesis, fatty acid oxidation and glycolysis. Increased energy requirements have been shown to increase the expression of PGC-1α (4). The high expression of PGC-1α can also be followed by its increased activity, which has been shown to cause an increase in mitochondrial mass and function (5). The function of mitochondria can be seen through the activity of succinate dehydrogenase (SDH) enzyme, an enzyme of the tricarboxylic acid (TCA) cycle, which is found in mitochondria (6).

Hypoxia-inducible factor-1α (HIF-1α), a protein that undergoes stabilisation in hypoxic conditions, is thought to regulate the expression of cytoglobin (CYGB). CYGB is known to play a role in oxygen supply, inhibit cell death and scavenge reactive oxygen species (ROS). Therefore, CYGB has a protective effect on oxidative stress in hypoxic conditions. Several studies have shown that there is a binding site for the HIF-1α protein on the CYGB gene promoter (7). This is consistent with our previous study, which found that CYGB is regulated by HIF-1α (8).

Our previous study proved that the administration of ibuprofen aimed to inhibit HIF-1α inhibited CYGB expression and also keloid fibroblast cell proliferation (9).

Excessive proliferation of keloid fibroblasts requires an adequate amount of energy, which mostly comes from the oxidative phosphorylation process in the mitochondria. Hence, the process requires functioning mitochondria. In addition, it also requires sufficient oxygen, which is provided by CYGB. Hitherto, the role of CYGB in the metabolism of fibroblasts in keloid has not been reported. We therefore hypothesised that inhibition of CYGB expression would inhibit mitochondrial function. The aim of this study was to analyse the mitochondrial function on the inhibition of CYGB with small interfering ribonucleic acid (siRNA) through measurement of PGC-1α expression and the activity of the SDH enzyme.

## Methods

### Materials

The materials used in the study were CYGB (human) siRNA, siRNA (−) control, siRNA transfection medium (sc-36868, Santa Cruz Technology, Dallas, TX, USA) and siRNA transfection reagent (Santa Cruz Technology), Dulbecco’s Modified Eagle’s Medium (DMEM) low glucose (Gibco^™^, Fisher Scientific, UK), 1% penicillin/streptomycin (Sigma-Aldrich, St Louis, MO, USA), 1% Fungizone (Gibco^™^, New York, NY, USA), 10% foetal bovine serum (FBS) (Gibco^™^) and Tryple Select (Gibco^™^) and a 24-well culture plate for fibroblast culture (Corning® Costar®, USA). The reagent used for total RNA isolation was Tripure Isolation Reagent (Promega, WI, USA) and for the isolation of protein, the Ripa Buffer 10X Protein (Abcam, Cambridge, UK). The SensiFAST SYBR No-ROX Kit was used for mastermix PCR (Bioline, London, UK), the enzyme-linked immunosorbent assays (ELISA) kit for human’s CYGB (Elabscience, UK) and for PGC-1α (Elabscience, UK) and the SDH Assay Kit (colorimetric) (Abcam, ab228560).

### Study Design

This study was an experimental study carried out on a keloid fibroblast cell culture derived from our previous study (10), which had been frozen at −80 °C. After the thawing process, a culture was grown on low-glucose DMEM medium. The sample came from three individual keloid patients who were each allocated to one of three study groups, namely: i) group without treatment; ii) group transfected with siRNA (+) CYGB and iii) group transfected with siRNA (−) CYGB. The study was conducted in the laboratory of the Department of Biochemistry & Molecular Biology, Faculty of Medicine Universitas Indonesia (FMUI), the Center of Hypoxia & Oxidative Stress Studies, FMUI and Indonesia Human Virology & Cancer Biology, FMUI, from July to December 2018.

### Cell Culture

Keloid fibroblast and preputium fibroblast from our previous study (10), which had been stored at −80 °C were replanted and grown in low-glucose DMEM growth medium with 10% FBS, 1% penicillin/streptomycin and 1% Fungizone. Cell growth was observed after 48 h–72 h of incubation at 37 °C, 95% O_2_, 5% CO_2_ in a humidified incubator.

### Transfection of siRNA

The keloid fibroblast cells were transfected with siRNA (+) CYGB (sc-36868) at a dose of 20 pmol (10) to silence CYGB using siRNA transfection medium. Initially, 6 × 10^4^ fibroblast cells were grown in triplicate on a 12-well plate. After 24 h incubation, transfection was performed. siRNA (+) CYGB was used in the treatment group (group 2) and siRNA (−) in the control group (group 3). The sequence target of the CYGB siRNA (+) is 5′-GGA GGA AUC CCU GAC UCA A-3′ and the sequence target for CYGB siRNA (−) is 5′-GAG CAG UCC CAU CGA UAG A-3′. Fibroblast cells were then incubated for 18 h, with addition of the medium after 6 h of incubation. After 24 h, the medium was replaced with the new medium, and fibroblast cells were incubated for 48 h and harvested for the assay. The expression of CYGB mRNA was measured using quantitative real time polymerase chain reaction (qRT-PCR) to evaluate the silencing effect of CYGB siRNA (+).

### Isolation of Total RNA

Total RNA was isolated from the three groups (CYGB siRNA (+); CYGB siRNA (−) and control) using TriPure^TM^ isolation reagent (Roche) according to the manufacturer’s protocol. RNA concentration and purity were measured using a spectrophotometer.

### Protein Isolation

After treatment, keloid fibroblast cells from CYGB siRNA (+), CYGB siRNA (−) and control were harvested. Keloid fibroblasts were lysed using RIPA buffer (Sigma-Aldrich) according to the manufacturer’s protocol. Total protein concentration was measured using a spectrophotometer. This lysate was used for determination of CYGB and PGC-1α protein.

### Quantitative RT-PCR to Measure Expressions of CYGB and PGC-1α

Fifty ng of RNA was used per 10 mL of reverse transcription (RT) reaction. An assay was performed using LightCycler^®^ 480 RT-PCR with SensiFAST^TM^ SYBR^®^ No-ROX One-Step Kit (Cat. No. BIO-72001, Bioline (Aust.) Pty Ltd, Australia).

Primers for CYGB were Forward 5′-CAGTTCAAGCACATGGAGGA-3′ and Reverse 5′-GTGGGAAGTCACTGGCAAAT-3′ (10); for PGC-1 alpha, the primers were Forward 5′-CTGACCACAAACG ATGACC-3′ and Reverse 5′-GAACAAATCTGCCCCTGC-3′ (11); and for 18S rRNA, the primers were Forward 5′-AAACGGCTACCACATCCAA-3′ and Reverse 5′-CCTCCAATGGATCCTCGTTA–3′ (10).

The qRT-PCR procedure was as follows: synthesise cDNA 5 min at 45 °C; reverse transcriptase inactivation (polymerase activation) for 2 min at 95 °C; PCR cycles carried out for 40 cycles for 5 sec at 95 °C for the denaturation stage; 10 sec at 60 °C for the CYGB, PGC-1α and 18S rRNA genes (through optimisation) for annealing; and 5 sec at 72 °C for elongation, the steps being added with 2 min for incubation.

Relative expressions of CYGB and PGC-1α were calculated using the Livak formula.

### Determination of CYGB and PGC-1α protein

CYGB and PGC-1α were measured using a sandwich enzyme-linked immunosorbent assay according to the manufacturer’s protocol. Determination of CYGB/PGC-1α protein was initiated by adding 100 μL of the sample (protein lysate) into each well. Samples were incubated for 1 h 30 min at 37 °C. The contents of the well were discarded and, anti-CYGB antibody/anti-PGC-1α antibody, which had been labelled with biotin (Biotinylated Detection Ab, Sigma-Aldrich), was added and incubated for 1 h at 37 °C. After that, the entire solution was discarded and each well was washed with 350 μL of washing buffer three times. Then, all the solution in the wells was discarded and streptavidin conjugated with peroxidase (avidin-HRP conjugated) was added and incubated for 30 min at 37 °C. In the next step, all the liquid in each well was discarded again and washed with washing buffer five times. Next, 90 μL of the substrate was added and incubated for 15 min at 37 °C. At the final stage, the stop solution was added to each well without removing the contents of the well, and the absorption of coloured compound was determined as 450 nm, using an ELISA reader.

### Activity of SDH Enzyme (Abcam, ab228560)

SDH enzyme converts succinate to fumarate and transfers the electron to an artificial electron acceptor (Probe), which changes the colour from blue to colourless (depending upon the sample enzymatic activity). The activity of SDH enzyme was measured using a colorimetric method on a 600 nm wavelength.

Altogether 500,000 cells were homogenised with 100 μL SDH enzyme buffer assay, then centrifuged at 10,000 g for 5 min. The supernatant obtained was transferred to a new tube. Then, 96-well plates were prepared for the SDH enzyme activity assay. Each well was filled with 50 μL SDH enzyme reaction mix with a composition of 46 μL SDH enzyme assay buffer, 2 μL SDH enzyme probe and 2 μL SDH enzyme mix substrate. Twenty-five millilitre of samples were added into each well and mixed with the SDH enzyme buffer assay to a volume of 50 μL (2× dilution). The diluted sample was added to each well and then 50 μL SDH enzyme reaction mix was slowly added, so that the final volume was 100 μL of a homogeneous mixture. The absorbance was measured at a wavelength of 600 nm for 30 min with an interval every 3 min.

### Statistical Analysis

Data were presented as mean (standard error of the mean [SEM]) of the triplicate experiment. Statistical analysis was further determined using an unpaired *t*-test for normal distribution data and the Mann-Whitney test for abnormal distribution data. Statistical significance was considered if *P* < 0.05.

## Results

### Expression of CYGB mRNA and Protein

**Figure 1:**
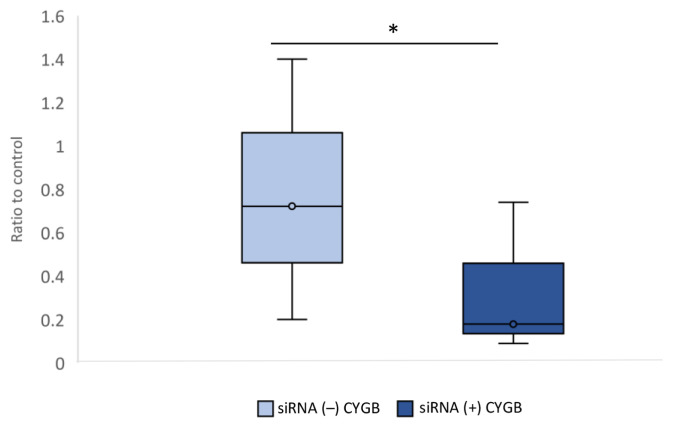
CYGB mRNA expression on transfection with CYGB siRNA (+) compared to siRNA (−). There was a decrease in CYGB mRNA expression in siRNA (+) CYGB compared to siRNA (−) CYGB (*P < 0.001; Mann-Whitney U test). Data normalised to control

Analysis of CYGB mRNA expression was performed on siRNA transfected keloid fibroblasts. Figure 1 shows the expression of CYGB mRNA in siRNA (+) and siRNA (−). CYGB mRNA expression in the CYGB siRNA (+) group (median = 0.179 [0.09–0.74]) was found to be significantly lower than the CYGB siRNA (−) group (median = 0.722 [0.2–1.4]; *P* < 0.001; Mann-Whitney U test).

**Figure 2:**
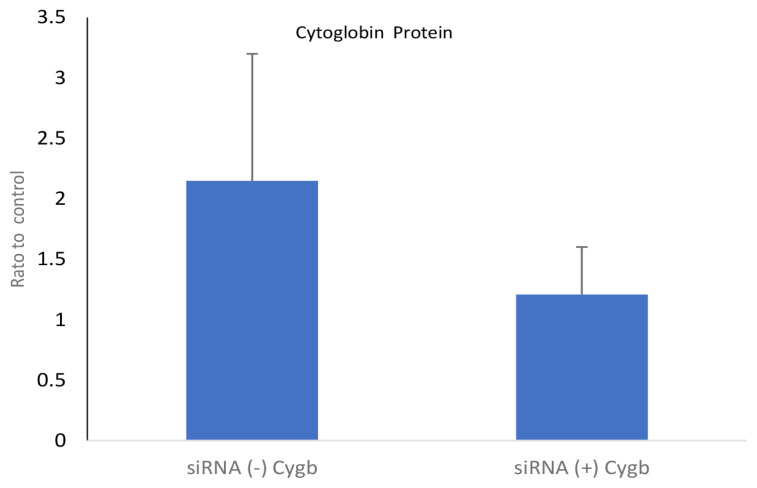
The levels of CYGB protein in cultured keloid fibroblasts on transfection with CYGB siRNA (+). CYGB protein levels were not significantly lower (1.208 [0.227]) on transfection with siRNA (+) CYGB compared to CYGB siRNA (−) (2.149 [0.606]); mean difference (95% CI) = 0.941 (−0.857, 2.738); *P* = 0.220; *t*-test. Data normalised to control

Inhibition expression of CYGB by siRNA (+) (1.208 [0.227]) in keloid fibroblasts also decreased the CYGB protein level, compared to CYGB siRNA (−) (2.149 [0.606]); mean difference (95% CI) = 0.941 (−0.857, 2.738; *P* = 0.220; *t*-test), as shown in Figure 2.

### Analysis of PGC-1α mRNA and Protein

**Figure 3:**
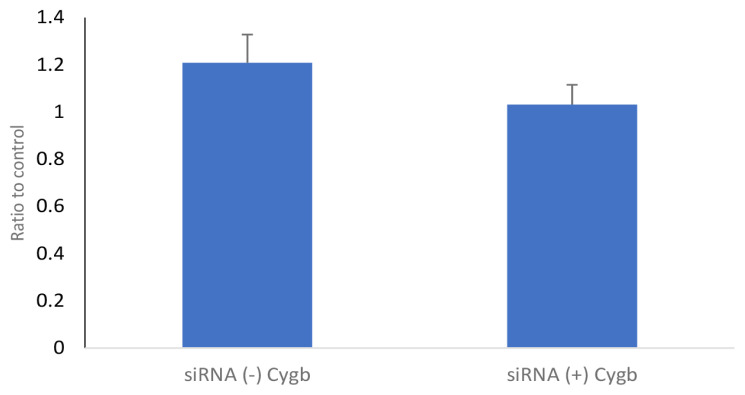
Expression of PGC-1α mRNA in keloid fibroblasts culture on transfection with CYGB siRNA (+). PGC-1α mRNA expression in CYGB siRNA (+) was not significantly lower (1.031 [0.084]) than CYGB siRNA (−) (1.208 [0.120]); mean difference (95% CI) = 0.177 (−0.118, 0.472); *P* = 0. 234; *t*-test. Data normalised to control

The analysis of PGC-1α mRNA expression on CYGB inhibition of keloid fibroblasts is shown in Figure 3. The results showed that PGC-1α mRNA expression in the CYGB siRNA (+) group (1.031 [0.084]) was lower, though not significantly, than the CYGB siRNA (−) group (1.208 [0.120]); mean difference (95% CI) = 0.177 (−0.118, 0.472; *P* = 0.234); *t*-test.

**Figure 4:**
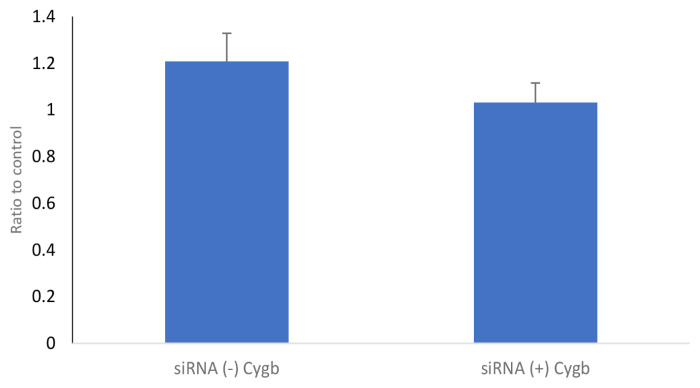
Protein levels of PGC-1α in keloid fibroblasts culture on transfection with CYGB siRNA (+). PGC-1α protein levels in CYGB siRNA (+) were not significantly lower (1.989 [0.181]) than siRNA (−) (2.538 [0.401]); mean difference (95% CI) = 0.549 (−0.431, 1.529); *P* = 0.240; *t*-test. Data normalised to control

The PGC-1α protein levels in the keloid fibroblasts culture on transfection with CYGB siRNA (+) are shown in Figure 4. The results showed that PGC-1α protein levels in the CYGB siRNA (+) group (1.989 [0.181]) were insignificantly lower than the CYGB siRNA (−) group (2.538 [0.401]); mean difference (95% CI) = 0.549 (−0.431, 1.529); *P* = 0.240; *t*-test.

### Analysis of Succinate Dehydrogenase Enzym Activity

**Figure 5:**
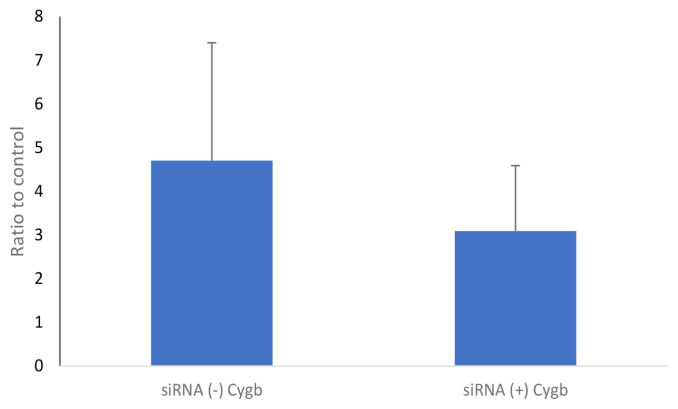
Activity of SDH enzyme in keloid fibroblasts culture on transfection with CYGB siRNA (+). Activity of SDH enzyme was lower (3.092 [0.863]), though not significantly so, in the CYGB siRNA (+) group compared to the CYGB siRNA (−) group (4.699 [1.559]); mean difference (95% CI) = 1.607 (−3.341, 6.554); *P* = 0.418; *t*-test. Data normalised to control

The activity of SDH enzym in the keloid fibroblast culture on transfection with CYGB siRNA (+) is shown in Figure 5. These results showed that SDH enzyme activity was lower, though not significantly, in the CYGB siRNA (+) group (3.092 [0.863]) compared to the CYGB siRNA (−) group (4.699 [1.559]; mean difference (95% CI) = 1.607 (−3.341, 6.554); *P* = 0.418; *t*-test).

## Discussion

The results showed that average CYGB mRNA and CYGB protein expressions were lower in the CYGB siRNA (+) group compared to the CYGB siRNA (−) group (Figs. 1 and 2). These results indicate that the lower expression of CYGB mRNA is due to the ability of the CYGB siRNA (+) molecule to bind specifically to the target (CYGB), thereby preventing complementary bonds and causing specific mRNA degradation and reduced CYGB protein. It is known that siRNA is a molecule composed of specific base pairs that correspond to the target gene. The binding of siRNA to the target gene is able to reduce the expression of the target mRNA, by cutting (degrading) the target mRNA (12).

Keloids are proven to be in a state of hypoxia, indicated by increased expression of HIF-1α. Our previous study demonstrated that CYGB expression in keloid fibroblast cultures is affected by HIF-1α (9). Inhibition of HIF-1α protein by ibuprofen caused a decrease in CYGB expression and decreased the proliferation of keloid fibroblasts. It has not yet been explained whether the decrease in proliferation is due to direct inhibition by HIF-1α or indirectly mediated by CYGB (9). Other studies have suggested that decreased HIF-1α expression causes cells to fail to adapt to hypoxia conditions, which leads to cell stress and changes the CYGB properties from tumour suppressors to oncogenes and increases proliferation (13)

The effect of CYGB inhibition by the siRNA molecule was proven in our previous study. Specifically, the inhibition of CYGB expression by siRNA caused an increase in reactive oxygen species (ROS) (10). However, proliferation of fibroblasts showed only a slight and insignificant increase (14). Research on the effect of CYGB on cell proliferation has yielded different results. Chen’s study revealed that, in ovarian cancer cell line treated with CYGB siRNA resulted in an increase in cell proliferation (15).

PGC-1α mRNA and protein expression in fibroblasts transfected with CYGB siRNA (+) tended to be lower than in fibroblasts transfected with CYGB siRNA (−) (Figures 4 and 5). The decrease in PGC-1α protein levels is thought to be a result of suppression of CYGB expression in keloid fibroblasts. According to Le Bleu et al. (16), PGC-1α mediates mitochondrial biogenesis and oxidative phosphorylation in cancer cells. Tohme et al. (17) also stated that hypoxia mediates mitochondrial biogenesis in hepatocellular carcinoma. In keloids, hypoxia is known to occur owing to increased proliferation of fibroblasts for the process of fibrogenesis. Therefore, if oxygen availability is limited because of an inhibition of CYGB expression, it will also affect the expression of PGC-1α.

In this study, we also measured the activity of SDH enzyme, which is an enzyme of the citric acid cycle and plays a role in the oxidative phosphorylation process in the mitochondria. The results showed that SDH enzyme activity in fibroblasts transfected by siRNA (+) CYGB was lower than siRNA (−) CYGB, although not significantly so. SDH enzyme is a unique enzyme in the oxidative phosphorylation process. It is a part of the electron transport chain complex and also acts in the oxidation of succinate to fumarate in the citric acid cycle (18). A decrease in SDH enzyme activity will have an impact on increasing succinate levels in fibroblasts, which will in turn increase the intracellular levels of ROS (19). The decrease in SDH enzyme activity is in line with the decrease in PGC-1α in fibroblasts transfected with CYGB siRNA. A decrease in mitochondrial biogenesis, which is characterised by a decrease in PGC-1α, will affect the maturation process of the SDH enzyme that takes place in the inner membrane of the mitochondria. Hence, impairment of mitochondrial biogenesis will certainly also interfere with the maturation of the enzyme. The insignificant decrease in expression of PGC-1α and SDH enzyme activity is likely due to keloid metabolism, which does not depend entirely on oxidative phosphorylation, because keloids rely more on energy sources from anaerobic glycolysis (20).

## Conclusion

We concluded that mitochondrial function is impaired in the inhibition of CYGB expression with siRNA in keloid fibroblasts, as indicated by decreased PGC-1α expression and the activity of the SDH enzyme, both of which are markers of mitochondrial function. An understanding of the metabolic aspects of keloid fibroblasts may be used in keloid treatment and possibly also in the therapeutic approach for cancer cells, based on their similarity in metabolism.
